# Monitoring of oxidative and metabolic stress during cardiac surgery by means of breath biomarkers: an observational study

**DOI:** 10.1186/1749-8090-2-37

**Published:** 2007-09-18

**Authors:** Florian Pabst, Wolfram Miekisch, Patricia Fuchs, Sabine Kischkel, Jochen K Schubert

**Affiliations:** 1 Department of Anesthesia and Intensive Care, University of Rostock, Schillingallee 35, 18057 Rostock, Germany

## Abstract

**Background:**

Volatile breath biomarkers provide a non-invasive window to observe physiological and pathological processes in the body. This study was intended to assess the impact of heart surgery with extracorporeal circulation (ECC) onto breath biomarker profiles. Special attention was attributed to oxidative or metabolic stress during surgery and extracorporeal circulation, which can cause organ damage and poor outcome.

**Methods:**

24 patients undergoing cardiac surgery with extracorporeal circulation were enrolled into this observational study. Alveolar breath samples (10 mL) were taken after induction of anesthesia, after sternotomy, 5 min after end of ECC, and 30, 60, 90, 120 and 150 min after end of surgery. Alveolar gas samples were withdrawn from the circuit under visual control of expired CO_2_. Inspiratory samples were taken near the ventilator inlet. Volatile substances in breath were preconcentrated by means of solid phase micro extraction, separated by gas chromatography, detected and identified by mass spectrometry.

**Results:**

Mean exhaled concentrations of acetone, pentane and isoprene determined in this study were in accordance with results from the literature. Exhaled substance concentrations showed considerable inter-individual variation, and inspired pentane concentrations sometimes had the same order of magnitude than expired values. This is the reason why, concentrations were normalized by the values measured 120 min after surgery. Exhaled acetone concentrations increased slightly after sternotomy and markedly after end of ECC. Exhaled acetone concentrations exhibited positive correlation to serum C-reactive protein concentrations and to serum troponine-T concentrations. Exhaled pentane concentrations increased markedly after sternotomy and dropped below initial values after ECC. Breath pentane concentrations showed correlations with serum creatinine (CK) levels. Patients with an elevated CK-MB (myocardial&brain)/CK ratio had also high concentrations of pentane in exhaled air. Exhaled isoprene concentrations raised significantly after sternotomy and decreased to initial levels at 30 min after end of ECC. Exhaled isoprene concentrations showed a correlation with cardiac output.

**Conclusion:**

Oxidative and metabolic stress during cardiac surgery could be assessed continuously and non-invasively by means of breath analysis. Correlations between breath acetone profiles and clinical conditions underline the potential of breath biomarker monitoring for diagnostics and timely initiation of life saving therapy.

## Background

Prevention and treatment of organ dysfunction or organ failure after cardiac surgery are crucial issues in post operative critical care medicine. Organ damage and poor patient outcome may be caused by ischemia/reperfusion, oxidative [1] or metabolic stress [2] during surgery and extracorporeal circulation.

Early recognition of these pathological conditions is the only way to minimize organ damage. All methods used so far (e.g. Swan-Ganz-Catheter, PICCO, Doppler based cardiac output measurements, laboratory parameters) are time and money consuming, invasive [3] and in the case of lab parameters often not fast enough and not immediately available at the bedside. In addition, sensitivity and specificity of these diagnostic methods are not satisfactory as far as early recognition and prevention of metabolic or oxidative stress and ischemia/reperfusion injury are concerned [4,5]. As a consequence, diagnosis happens too late and organ damage cannot be prevented. As these conditions are responsible for a major part of multiple organ failure one may expect that patients' outcome could be significantly improved through early recognition and consecutive induction of therapy [6].

Numerous studies have demonstrated a close correlation between clinical conditions and the exhalation of volatile biomarkers [7,8]. Some of these volatile substances are excreted into breath within minutes of their formation in tissues. Exhaled concentrations of these compounds can, therefore, be used to detect pathological conditions in the body at an early stage [9]. As breath tests are completely non invasive they can be performed repeatedly or continuously without any burden to the patient.

This study was intended to assess the impact of heart surgery with extracorporeal circulation (ECC) onto breath biomarker profiles. In detail, the following issues were to be addressed:

1. Do concentrations of exhaled volatile substances mirror clinical conditions during heart surgery?

2. When in the course of surgery do metabolic or oxidative stress and ischemia/reperfusion injury occur?

3. Which are clinical parameters that might induce detrimental changes in the body?

For that purpose the volatile lipid peroxidation marker pentane was determined in patients' exhaled air. In addition, exhaled acetone and isoprene concentrations were analyzed to investigate the effects of surgery and ischemia/reperfusion onto dextrose and cholesterol metabolism, metabolic stress and lipolysis.

## Methods

After approval by the institutional board and after having obtained informed consent 24 patients having heart surgery with ECC were enrolled into the study. All patients had to be older than 18 years, and all patients had indwelling pulmonary artery catheters for reasons of underlying diseases or kind of surgery. 13 patients were female and 11 male, the mean age was 69 years (range 55 – 80),16 suffered from coronary heart disease (CAD) and were scheduled for coronary artery bypass grafting (CABG), 2 of these had additional aortic valve stenosis, one had left ventricular aneurysm and one had stenosis of carotid artery. In these patients surgery was extended according to diagnoses. Four patients had aortic valve replacement, two had mitral valve replacement and two had combined mitral and aortic valve replacement. In 18 patients cardiac surgery was performed for the first time, 6 patients had recurrent surgery.

In each patient age, body mass index, medical history (diabetes, lipid disorders, COPD) and actual medications (statins, corticosteroids) were recorded. 12 patients suffered from diabetes. 7 of them were insulin dependent. 4 patients had COPD.

In addition, the following serum parameters were determined: K^+^, Na^+^, Ca^2+^, PT, PTT, hemoglobin, hematocrit, platelets, leucocytes, bilirubine, urea, dextrose, CRP, P_cap_O_2_, P_cap_CO_2_. Before surgery patients underwent thorough cardiac diagnostics, including determination of ejection fraction.

Preparation for surgery, anesthesia and perioperative vital data monitoring (5 channel ECG, pulse oximetry, arterial blood pressure measurement, 7F pulmonary artery catheter (Arrow, Erdingen, Germany)) was done in the same standardized way for any patient. Before anesthesia was begun patients got 250 mg of methyl prednisolone intravenously. Anesthesia was induced with sufentanil, etomidate and cis-atracurium and maintained with midazolam, sufentanil and cis-atracurium. Ventilation during anesthesia was performed in volume controlled mode. Respiratory rate and tidal volume were adjusted in the way that normocapnia was achieved. 22 patients got 2 g cefazoline, 6 patients other antimicrobial drugs as perioperative prophylaxis.

For skin incision always a scalpel was used. Electrocautery was employed for dissection of subcutaneous tissues and for bleeding control. When patients underwent cardiac surgery for the first time sternotomy was performed by means of a jigsaw, in the case of recurrent surgery an oscillating saw was used. After sternotomy all patients got 2 millions IU of aprotinine and 400 IU/kg of heparine intravenously before start of cardio pulmonary bypass (CPB). For CPB continuous, non pulsatile flow was employed, oxygenation was achieved by means of uncoated membrane oxygenators. Priming of heart and lung machine consisted of 1000 mL of gelatine solution, 500 mL of normal electrolyte solution, 5000 IE heparine, 250 mg methyl prednisolone and 1000 mg vitamin C.

During surgery duration of aortic clamping and CPB, volume of cardioplegic solution used, volume of hemodilution and infusions, diuresis and minimum body temperature and dose of catecholamines at the moment of weaning from CPB were recorded.

After surgery patients were transferred to the intensive care unit (ICU). All patients were sedated and mechanically ventilated beyond the end of the last breath gas analysis performed within the study. On arrival in the ICU, an arterial blood gas analysis was done, and hemoglobin, hematocrit, platelets, creatinine, dextrose, K^+^, Na^+^, Ca^2+ ^were determined in patients' blood. About four hours after the end of surgery blood was taken again to determine creatinine kinase (CK), CK-myocardium/brain (CK-MB) and troponine T (TNT). Diuresis, dose of catecholamines and transfusion of blood or blood products during stay in ICU were recorded.

### Breath sampling

Breath samples were taken after induction of anesthesia, after sternotomy, 5 min after end of ECC, and 30, 60, 90, 120 and 150 min after end of surgery. For that purpose, a sterilized stainless steel T-piece and the measuring cuvette of a commercially available capnometer had been incorporated into the respiratory circuit near the endotracheal tube (ETT). Contamination of respiratory circuits with volatile anesthetics was meticulously excluded. Breath samples (10 mL) were drawn from the respiratory circuits into a gas tight syringe and transferred immediately into evacuated 20 mL glass vials. Alveolar breath samples were taken manually from the expiratory limb of the respiratory circuit by means of a gas tight syringe in a CO_2 _controlled way as described before [10-12].

For that purpose, the sensor of a fast responding mainstream CO_2 _analyzer (Capnogard, Novametrix Medical Systems Inc., Wallingford, CT, USA) was inserted into the respiratory circuit near the ETT. Alveolar gas samples were withdrawn from the circuit under visual control of expired CO_2 _in the way that gas collection only took place during the alveolar phase of expiration. Inspiratory samples were taken near the ventilator inlet. The access to the respiratory circuit was realized through sterilized stainless steel T-pieces. At least two samples for each measurement were collected. Inspired samples were withdrawn from the inspiratory limb of the ventilator tubing [13]. Although it was known from pre-study experiments that samples in the vials remain stable for at least 8 h, preconcentration and analysis was begun immediately after sampling.

### Preconcentration and analysis of volatile substances

Volatile substances in breath were preconcentrated by means of solid phase micro extraction (SPME) [14-16]. Preconcentration and desorption of the volatile organic compounds were done automatically by means of a SPME autosampler (CTC Multi Purpose Sampler, PAL, Zwingen, Switzerland). Before the SPME procedure, the PDMS/Carboxen-coated fiber was pre-treated in the injection port of a gas chromatograph at 285°C for 30 min. Internal standard (2,3-dimethylbutadiene) was added through the septum by means of a micro syringe. The vial was vortexed for 5 min at 40°C in the heating block of the CTC autosampler. Then, the syringe needle of the SPME device was inserted into the vial for 5 min. The needle containing the SPME fiber was withdrawn and introduced into the port of a capillary gas chromatograph (Varian, Walnut Creek, CA, USA). Port temperature was 280°C. The SPME device was held in the port for 2 min to allow complete desorption of the components.

Substances were separated on a 30 m (0.32 mm ID.) Porabond Q column (Varian/Chrompack, Middleburg, The Netherlands), detected and identified by mass spectrometry (Saturn 2000, Varian, Walnut Creek, CA, USA) and quantified via calibration curves. Precision, variation and limits of detection of SPME procedures have been extensively studied in previous work [11,16-19]. Limits of detection (LODs) for the analysis of isoprene, pentane and acetone in exhaled gas were 0.01, 0.02 and 0.1 nmol/L. Intraday variation was < 5% relative standard deviation (RSD) (n = 8) for all substances.

### Statistical analysis

Results are given as medians and 25–75% percentiles or as means and 95% confidence intervals, as appropriate. Correlation between different variables was assessed by means of Spearman correlation coefficients for not parametric data. Multiple comparisons between data from different measurements during and after surgery were done by means of One Way Repeated Measures Analysis of Variance for normally distributed values or by means of Friedman Repeated Measures Analysis of Variance on Ranks for non parametric data. The Student-Newman-Keuls test was used for post-hoc analysis. Comparison between only two patient groups was done by Wilcoxon signed rank test.

In order to render results comparable between different patients exhaled substance concentrations were normalized to respective concentrations at 120 min after end of surgery (C_i_/C_120_). By means of that normalization effects of inspiratory substance concentrations were also eliminated as each patient served as his own control.

P < 0.05 was considered to be statistically significant. For statistical testing the computer program SPSS 11.0 for Windows was used.

## Results

Inspired and mean exhaled substance concentrations are shown in table 1. Median normalized exhaled substance concentrations measured at different times during and after surgery were compared to each other. In addition, correlation coefficients between exhaled substance concentrations and clinical parameters (serum dextrose, BMI, CK-MB/CK, CRP, CI, serum lactate, MAP, PAP, PCWP, TNT, duration of aortic clamping and ECC) were determined.

**Table 1:** Mean substance concentrations in nmol/L

**Substance**	**t1**	**t2**	**t3**	**t4**	**t5**	**t6**	**t7**	**t8**	**ins1**	**ins2**
Acetone	8.84	13.8	32.4	31.4	32.1	31.7	32.9	28.4	5.37	1.48
± SD	6.85	8.83	25.6	24.3	23.6	23.3	22.3	23.5	4.68	0.94
Pentane	0.33	0.40	0.18	0.11	0.11	0.12	0.13	0.13	0.13	0.10
± SD	0.43	0.47	0.16	0.12	0.10	0.12	0.15	0.17	0.20	0.14
Isoprene	3.66	5.51	5.11	2.68	2.88	3.49	3.23	3.14	3.66	5.51
± SD	4.26	5.66	5.75	2.36	2.71	4.64	3.81	3.32	4.26	5.66

t1 after induction of anesthesia, t2 after sternotomy, t3 5 min after end of ECC, t4 30 min, t5 60 min, t6 90 min, t7 120 min after surgery, t8 150 min after surgery. Ins1 inspired concentrations in the OR, ins 2 inspired concentrations in the ICU.

### Acetone

**Figure 1:**
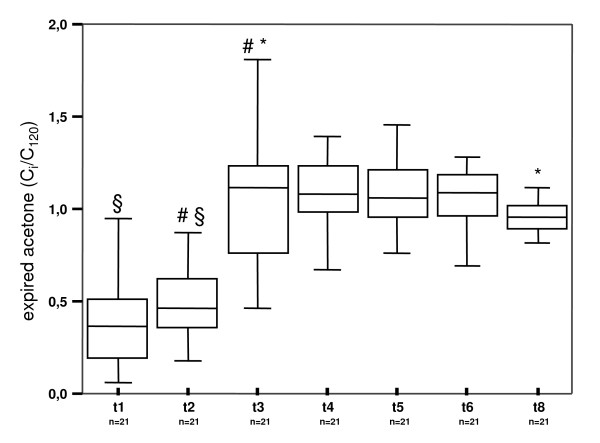
Exhaled acetone concentrations before, during and after surgery. T1 – t8 indicate time of breath gas testing in the patients. t1 after induction of anaesthesia, t2 after sternotomy, t3 5 min after end of extracorporeal circulation, t4 30 min, t5 60 min, t6 90 min and t8 150 min after end of surgery. Exhaled substance concentrations were normalized to respective concentrations at t7 120 min after end of surgery (C_i_/C_120_). N number of patients in which exhaled acetone was determined. #, § * indicate significant differences between median values.

Exhaled acetone concentrations increased slightly after sternotomy and markedly after end of ECC. Post operatively, concentrations were constant (Figure 1) on a higher level. Exhaled acetone concentrations exhibited positive correlation with serum C-reactive protein concentrations and with serum troponine-T concentrations (Table 2).

**Table 2:** Correlation of exhaled acetone concentrations (C_i_/C_120_) with clinical parameters

**Parameter**	**t1**	**t2**	**t3**	**t4**	**t5**	**t6**	**t8**
CRP before surgery	ns	p < 0.05	p < 0.01	p < 0.05	p < 0.01	ns	ns
CRP after surgery	ns	ns	ns	ns	p < 0.05	ns	ns
Serum lactate after surgery	ns	ns	ns	p < 0.05	ns	ns	ns
TNT after surgery	ns	ns	p < 0.05	p < 0.01	p < 0.05	ns	p < 0.05
Duration of aortic clamping	ns	ns	ns	p < 0.05	ns	ns	ns
Duration of ECC	ns	ns	ns	p < 0.05	p < 0.05	ns	ns

t1 after induction of anesthesia, t2 after sternotomy, t3 5 min after end of ECC, t4 30 min, t5 60 min, t6 90 min, t8 150 min after surgery. ns correlation not significant.

### Pentane

**Figure 2:**
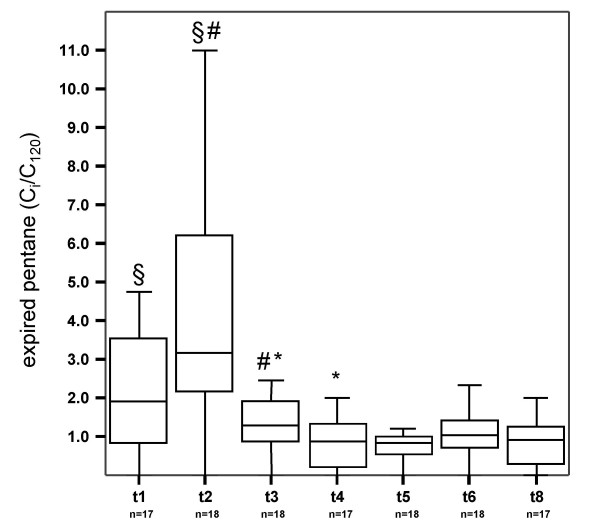
Exhaled pentane concentrations before, during and after surgery. T1 – t8 indicate time of breath gas testing in the patients. t1 after induction of anaesthesia, t2 after sternotomy, t3 5 min after end of extracorporeal circulation, t4 30 min, t5 60 min, t6 90 min and t8 150 min after end of surgery. Exhaled substance concentrations were normalized to respective concentrations at t7 120 min after end of surgery (C_i_/C_120_). N numbers of patients in which exhaled pentane was determined. #, § * indicate significant differences between median values.

Exhaled pentane concentrations increased markedly after sternotomy. After ECC exhaled pentane concentrations dropped below initial values. (Figure 2). Breath pentane concentrations exhibited correlations with serum creatinine (CK) levels. Patients with an elevated CK-MB (myocardial&brain)/CK ratio had also high concentrations of pentane in exhaled air (Table 3).

**Table 3:** Correlation of exhaled pentane concentrations (C_i_/C_120_) with clinical parameters

**Parameter**	**t1**	**t2**	**t3**	**t4**	**t5**	**t6**	**t8**
CK-MB/CK after surgery	ns	ns	p < 0.01	ns	ns	ns	ns

t1 after induction of anesthesia, t2 after sternotomy, t3 5 min after end of ECC, t4 30 min, t5 60 min, t6 90 min, t8 150 min after surgery. ns correlation not significant.

### Isoprene

**Figure 3:**
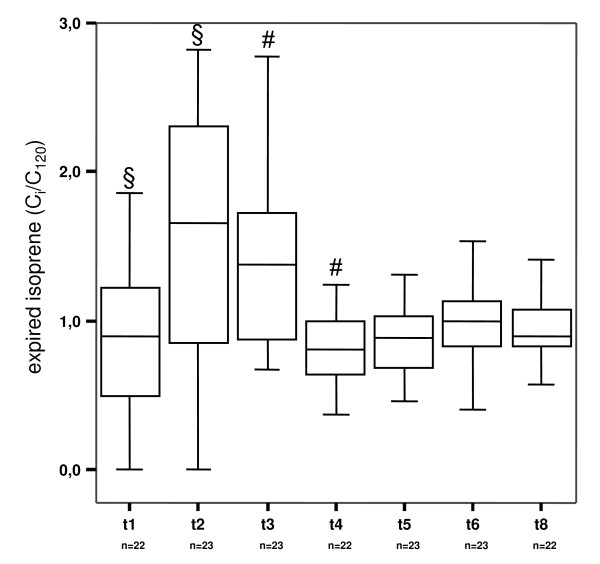
Exhaled isoprene concentrations before, during and after surgery. T1 – t8 indicate time of breath gas testing in the patients. t1 after induction of anaesthesia, t2 after sternotomy, t3 5 min after end of extracorporeal circulation, t4 30 min, t5 60 min, t6 90 min and t8 150 min after end of surgery. Exhaled substance concentrations were normalized to respective concentrations at t7 120 min after end of surgery (C_i_/C_120_). N numbers of patients in which exhaled isoprene was determined. #, § indicate significant differences between median values.

Exhaled isoprene concentrations raised significantly after sternotomy (p < 0,05) and decreased to initial levels at 30 min after end of ECC (Figure 3). Exhaled isoprene concentrations showed a correlation with cardiac output. Patients having high cardiac index exhaled more isoprene than those with low cardiac index (Table 4).

**Table 4:** Correlation of exhaled isoprene concentrations (C_i_/C_120_) with clinical parameters

**Parameter**	**t1**	**t2**	**t3**	**t4**	**t5**	**t6**	**t8**
BMI	ns	ns	P < 0,01	ns	ns	ns	ns
CK-MB/CK after surgery	ns	ns	ns	p < 0,05	p < 0,05	ns	ns
CI	ns	ns	P < 0,01	ns	ns	ns	p < 0,01

t1 after induction of anesthesia, t2 after sternotomy, t3 5 min after end of ECC, t4 30 min, t5 60 min, t6 90 min, t8 150 min after surgery. ns correlation not significant. BMI body mass index, CK-MB creatinine kinase myocardium/brain

## Discussion

Profiles of volatile biomarkers measured during and after cardiac surgery showed correlations with clinical conditions or clinical parameters. Acetone in breath mirrored metabolic stress, exhaled pentane concentrations increased during well defined surgical actions, exhaled isoprene showed a correlation to cardiac output. The profile of acetone exhalation parallels facts known from clinical studies on outcome and serum dextrose control [2].

Mean exhaled concentrations of acetone, pentane and isoprene determined in this study were in accordance with results published during recent years [7,9,20,21].

Exhaled substance concentrations showed considerable inter-individual variation, and inspired pentane concentrations sometimes had the same order of magnitude than expired values. This is the reason why, concentrations were normalized by the values measured 120 min after surgery. In this way, each patient served as his own control, effects of inter-individual variation and impact of inspired concentrations onto results were reduced.

Release of catecholamines results in an increased lipolysis and release of corticosteroids induces an increased dextrose turnover. Concentrations of ketone bodies are increased when dextrose metabolism is impaired and lipolysis is triggered. Eventually, acetone is generated through decarboxylation of acetoacetate produced through condensation of Acetyl-CoA. This may explain the increase in exhaled acetone concentrations after end of ECC when levels of catecholamines raise or are artificially raised in order to wean the patient from ECC. Positive correlation between exhaled acetone concentrations and time of ECC, time of aortic clamping and post operative TNT may confirm this hypothesis as an increase of these parameters can induce increasing catecholamine levels in the body. The same holds true for the correlation between pre-operatively measured CRP and exhaled acetone concentrations as presence of SIRS or other inflammation can cause an increased need for endogenous or therapeutically applied catecholamines.

Although acetone concentrations were not directly correlated to serum dextrose levels, profiles of acetone concentrations in breath parallel facts known from clinical studies done in the early 2000s. Van den Berghe et al showed that increased blood dextrose levels occurring post-operatively were linked to poor patient outcome that could be improved when dextrose concentrations were controlled by means of insulin therapy [2]. The missing correlation between exhaled acetone concentrations and serum dextrose levels is due to the fact that acetone production does not directly depend on dextrose concentrations [22], that ketone generation is linked to lipolysis and that insuline therapy was already started in some of the patients of our study at the time of measurement. Nevertheless, acetone in breath apparently mirrored aspects of metabolic stress during heart surgery. As this volatile biomarker can be determined easily exhaled acetone could be used to recognize these pathological conditions and to prevent patients from being damaged by them.

Exhaled pentane concentrations only increased after sternotomy. Most probably this was due to the high extent of oxidative stress induced through the action of the saw and the electrocautery. Andreoni et al [23] observed the same phenomenon when they determined exhaled ethane concentrations during CABG. In an animal model they demonstrated that ethane generation during sternotomy was mainly due to electrocautery. In addition, as sternotomy happens early in the course of cardiac surgery its effects were not blunted by the application of corticosteroids, which take a certain time to develop antiphlogistic effects.

During ischemia/reperfusion reactive oxygen species (ROS) are generated through the action of xanthine oxygenase [24]. Reactive oxygen species (ROS), such as superoxide anion O_2_^- ^or hydroxyl radical OH physiologically act as defense mechanism [25] and can potentially damage any cellular structure including DNA and RNA. Under healthy conditions, ROS activity is restricted to limited regions of external attack or inflammation and is well balanced by antioxidant protection of the body. In some diseased states, such as ischemia/reperfusion, the balance between ROS activity and protection may be impaired when antioxidant systems are overwhelmed or exhausted [26]. Uncontrolled ROS action is the reason for ischemia induced organ dysfunction (MODS) or organ failure (MOF) [27,28]. MODS and MOF are important reasons of morbidity and mortality after major abdominal or cardio-vascular surgery. ROS attack onto cell membranes produces n-alkanes ethane and pentane through peroxidation of ω-3 and ω-6 fatty acids, respectively. Steep increase and immediate decrease of exhaled pentane concentrations after sternotomy indicate that pentane acts as a fast and direct biomarker. Correlation of exhaled pentane concentrations measured after end of ECC and CK/CK-MB ratio measured a few ours later underlines the potential usefulness of exhaled pentane for early recognition of oxidative stress and ischemia/reperfusion injury.

Surprisingly, pentane concentrations were not increased after ECC. Most probably, ECC induced ischemia/reperfusion injury was blunted by corticosteroid and antioxidant (vitamin C) medication given to the patients prior to surgery. The effects of antioxidants such as vitamin E onto breath alkane concentrations has already been observed by others [7,29].

Increased exhaled isoprene concentrations in myocardial infarction have been attributed to activation of neutrophiles in the ischemic myocardium [30,31]. The same mechanism may be responsible for the increase of isoprene levels in breath observed in our study. Decreasing substance concentrations after surgery may then possibly be due to sedation and analgesia [32,33]. However, the positive correlation between exhaled isoprene concentrations and cardiac output suggests a completely different mechanism, which can explain both effects. As cardiac output was higher after sternotomy and directly after ECC than during the postoperative phase, the effects described above may simply have been caused through changes of cardiac output.

Due to technical and methodological restrictions this study exhibits some flaws. As ppb (nmol/L) levels of ethane cannot be captured by SPME exhaled ethane concentrations were not determined. Knowing the results of Andreoni et al [23] the comparison between exhaled ethane and pentane concentrations could have been interesting. As the number of patients was small, especially with respect to multiple statistical comparisons, the power of clinical conclusions drawn from these results is limited. For the same reasons, potential effects of minute ventilation and cardiac output onto results could not be analyzed in detail. In order to identify the effects of vitamin C and corticosteroids onto exhaled pentane concentrations, a control group without this medication would have been necessary. As corticosteroids and vitamin C are standard treatment options in our institution, omitting these drugs could have prevented permission of the study.

## Conclusion

Oxidative and metabolic stress during cardiac surgery could be assessed continuously and non-invasively by means of breath analysis. Oxidative and metabolic stress occurred despite application of anti-inflammatory and anti-oxidant drugs. Correlations between breath acetone profiles and clinical conditions underline the potential of breath biomarker monitoring for diagnostics and timely initiation of life saving therapy.

## Competing interests

The author(s) declare that they have no competing interest.

## Authors' contributions

FP carried out patient recruitment, breath sampling in OR and ICU, record keeping, and participated in the interpretation of data. WM supervised the GC/MS analyses, was responsible for quality assurance and participated in statistical analysis. PF and SK performed the GC/MS analyses running the instruments and doing integration and quantification. PF helped to draft the manuscript. JS conceived of the study, participated in its design and coordination, supervised statistical analysis and drafted the manuscript.

All authors read and approved the final manuscript.
